# The use and impact of prehospital blood lactate measurements in acute non-traumatic patients: a systematic review

**DOI:** 10.1186/s13049-024-01310-1

**Published:** 2024-12-26

**Authors:** Louise Houlberg Walther, Hanne Beck Mieritz, Annmarie Touborg Lassen, Erika Frischknecht Christensen, Christian Backer Mogensen, Søren Mikkelsen, Anne Craveiro Brøchner

**Affiliations:** 1 The Prehospital Research Unit, Region of Southern Denmark, Odense University Hospital, J. B. Winsloews Vej 4, 5000 Odense C, Denmark; 2 Department of Regional Health Research, University of Southern Denmark, Odense, Denmark; 3 Department of Cardiothoracic Anesthesia and Intensive Care Medicine, Odense University Hospital, Odense, Denmark; 4 Department of Emergency Medicine, Odense University Hospital, Odense, Denmark; 5 Department of Clinical Research, University of Southern Denmark, Odense, Denmark; 6 Centre for Prehospital and Emergency Research, Aalborg University Hospital, Aalborg, Denmark; 7 Institute of Clinical Medicine, Aalborg University, Aalborg, Denmark; 8 Emergency Medicine Research Unit, Hospital Soenderjylland, University Hospital of Southern Denmark, Aabenraa, Denmark; 9 Department of Anesthesiology and Intensive Care, Lillebaelt University Hospital, Kolding, Denmark

**Keywords:** Lactate, Prehospital, Prognosis, Emergency

## Abstract

**Background:**

The prehospital use of blood lactate measurements is increasing. However, the test’s benefits have not been methodically evaluated in non-trauma patients. This study had three aims: (1) To assess the evidence of prehospital blood lactate measurements’ prognostic value in non-trauma patients, (2) to investigate to what extent the test changed early patient treatment, and (3) to evaluate the healthcare personnel’s attitude towards the test.

**Methods:**

MEDLINE, Embase, and Cochrane Central Register of Controlled Trials were systematically searched until Aug 26, 2023. Cohort and randomized controlled trials assessing ≥ 20 acute non-trauma patients with prehospital lactate measurements were included if they reported (1) prognostic outcomes such as short-term mortality or (2) changes in early patient treatments. All study designs were included to assess (3) the healthcare personnel’s opinion on prehospital lactate measurements. The risks of bias were assessed using the QUIPS tool, the Newcastle–Ottawa Scale, and the RoB-2. Study registration number CRD42020167169 (PROSPERO).

**Results:**

We screened 6028 study reports. We included 15 studies on (1) the prognostic value of prehospital lactate measurements. Elevated blood lactate levels were correlated to a higher short-term mortality risk in most of the studies but not in studies with out-of-hospital cardiac arrest (OHCA) patients. The 15 prognostic studies were all cohort studies with moderate or high risks of bias. Four studies investigated (2) early treatment changes. They found that the prehospital lactate measurement may have changed early treatment in sepsis patients. However, all four studies on treatment changes were at high risk of bias. Four studies were included on (3) the healthcare personnel’s attitude towards the lactate measurement. Evidence of the healthcare personnel’s opinion on prehospital lactate measurements was scarce.

**Conclusion:**

Most acute non-trauma patients with elevated prehospital lactate levels had increased risks of short-term mortality, except OHCA patients. Few studies suggested that measuring prehospital lactate levels could change early patient care, particularly in patients with suspected sepsis. The certainty of the evidence is low in this systematic review. The included studies were heterogeneous, and many had high risks of bias. Further studies are needed to investigate the impact of prehospital lactate measurements on patient care.

**Supplementary Information:**

The online version contains supplementary material available at 10.1186/s13049-024-01310-1.

## Introduction

Assessing acute patients can be challenging in emergency settings. Vital signs such as blood pressure, heart rate, and oxygen saturation may miss early deterioration [1]. Blood lactate measurement is a prognostic marker of poor outcomes [2]. Most research has focused on in-hospital lactate measurements [2–7]. It can be used for risk assessment in acute patients [2] and has been recommended in the SEPSIS-3 guidelines as a part of the initial patient assessment [8].

The assessment and treatment of the acute patient is initiated in the prehospital phase. Thus, a prehospital lactate measurement may support early decision-making in the prehospital setting and afterward in the emergency department. Results from in-hospital studies might not comply with acute patients in the prehospital setting, as the patients are assessed earlier in their course of disease. The in-hospital and prehospital patient populations may differ as well. As studies using prehospital lactate measurements emerge, a need for compiling the body of evidence in this field arises before a possible widespread implementation of prehospital lactate measurement is justified. The prognostic use of prehospital lactate measurements has been systematically reviewed in trauma patients in 2016 [9]. However, the evidence has not yet been assessed in other acute patient groups.

In-hospital blood lactate measurements are usually done on stationary equipment unfit for the prehospital environment. Several small point-of-care lactate meters have been approved for medical use in the last twenty years. These portable devices are better suited for use in ambulances and emergency helicopters and demonstrate good accuracy compared to lactate meters used in-hospital [10]. Still, new equipment should not be implemented without considering the pros and cons of this decision. A study suggested that lactate levels in risk stratification were superior to those of vital signs [1]. Later, another study showed that adding the lactate level to risk assessments enhanced risk stratification in acute prehospital patients [11]. Improved prognostication using prehospital lactate measurements could lead to more focused treatments being initiated earlier. In turn, this may improve patients’ outcomes. Still, it is essential that the lactate measurement is easy and fast to avoid unnecessary delays in the assessment of acute patients. Furthermore, it has not yet been shown that measuring the lactate level in acute patients increases diagnostic accuracy, e.g., in sepsis patients [12].

The healthcare personnel's attitudes toward lactate measurements could be one of the hurdles to a possible implementation in the prehospital setting, in which limited time and resources are important conditions [9].

Our systematic review aimed to assess the current evidence of prehospital blood lactate measurements in non-trauma patients. It includes three closely linked aims regarding the prognostic performance of prehospital lactate levels, possible changes in early treatment due to prehospital lactate measurements, and the healthcare personnel’s attitude toward using prehospital lactate measurements.

## Methods

This study was conducted according to a study protocol using the PRISMA-P guidelines [13]. The study was prospectively registered in PROSPERO (Registration number CRD42020167169) [14]. We reported our findings according to the PRISMA 2020 statement [15].

### Objectives

This systematic review identifies, summarizes, and analyzes studies of prehospital measurement of blood lactate in acute non-trauma patients. The review had the following three objectives, each resulting in the inclusion and analyses of different types of studies:

1. To evaluate the prognostic value of prehospital measured blood lactate on short-term mortality for acute non-trauma patients.
2. To evaluate whether the knowledge of the patients’ prehospital blood lactate level modified the clinicians’ early patient treatment in non-trauma patients.
3. To summarize to what extent the clinicians considered acute prehospital blood lactate measurement a valuable tool in early decision-making when treating non-trauma patients.

### Eligibility criteria

We included studies of measurement of blood lactate (both venous, arterial, and capillary analyses) in acute (i.e., not elective) non-traumatic patients of all ages assessed in the prehospital environment (and studies of clinicians who treated such patients). In Objectives 2 and 3, a clinician was defined as any healthcare professional providing acute care to patients, including EMS personnel, nurses, and physicians working with acute patients prehospital or in-hospital.

Studies were included regardless of language, year of dissemination, and report status. Studies with less than 20 patients were excluded in Objectives 1 and 2.

Studies were excluded if data were only available for trauma patients or if study results were not separated for trauma and non-trauma patients.

Core eligibility criteria were the same for the three aims.

#### Objective 1: The prognostic value of prehospital blood lactate

We included cohort studies that assessed the prognosis of patients with lactate assessments, including studies of the association between high and low lactate values and good or poor outcomes. We distinguished between studies in which lactate values were blinded to the health care personnel and those with unblinded values, but they were analyzed collectively. Studies were excluded if they assessed prehospital transfer patients who had already been evaluated/treated at the hospital.

Randomized controlled trials and cohort studies were included if they compared the difference in prognosis between patients with and without lactate measurements.

We were primarily interested in short-term mortality (i.e., up to 7 days, but up to 30 days was accepted if this was the only outcome reported). In-hospital mortality was also regarded as short-term mortality despite the fact that a part of the in-hospital mortality had occurred after 30 days. We combined in-hospital and ≤ 30 days mortality as short-term mortality in the analyses. Additional outcomes related to prognoses were also assessed: length of stay in the hospital or the intensive care unit (ICU), admission to ICU, and need for mechanical ventilation or vasopressors.

#### Objective 2: Changes in early patient care

We included effect studies assessing how knowledge of a patient’s prehospital blood lactate level had modified the clinician’s prehospital or in-hospital early treatment in acute non-trauma patients. Randomized controlled trials and cohort studies were included if they compared the treatments of patients with known and unknown lactate values.

Regarding patient treatment, we mainly implied fluid administration, blood transfusion(s), oxygen supply, and triage (altered receiving hospital or way of transportation to hospital). Other clinically relevant outcomes were also included if reported in the studies included in this review.

#### Objective 3: The clinicians’ opinions about prehospital lactate measurements

We included interview and questionnaire studies that explored whether and why the clinicians (i.e., health care personnel treating acute patients in-hospital or in the prehospital area) considered acute prehospital blood lactate measurement a valuable tool in early decision-making when treating non-trauma patients. Interview studies were included regardless of the number of interviewees.

We expected that this systematic review would find limited data investigating Objective 3. To enhance the comprehensibility of the data reporting, the data from this objective will not be reported further in the manuscript. Instead, see the Supplemental Files (Sect. 3 + Table S1) for results and information regarding this objective.

### Information sources

We searched three databases to find eligible studies: Medline (Ovid), Embase (Ovid), and Cochrane Central Register of Controlled Trials (Wiley). The last updated searches were done on August 26, 2023.

LHW conducted a backward citation search and searched the reference lists of three systematic review reports on blood lactate measurements in acute patients. Furthermore, a forward citation search was conducted using Google Scholar on November 29, 2023.

First authors of study reports presented as poster abstracts were contacted twice by email two weeks apart to retrieve supplemental study data. Correspondently, we contacted the authors of multiple study reports included in this systematic review to ensure that no study participants were included in our study more than once. Additionally, authors were contacted in case of any uncertainties.

### Search strategy

**Fig. 1:**
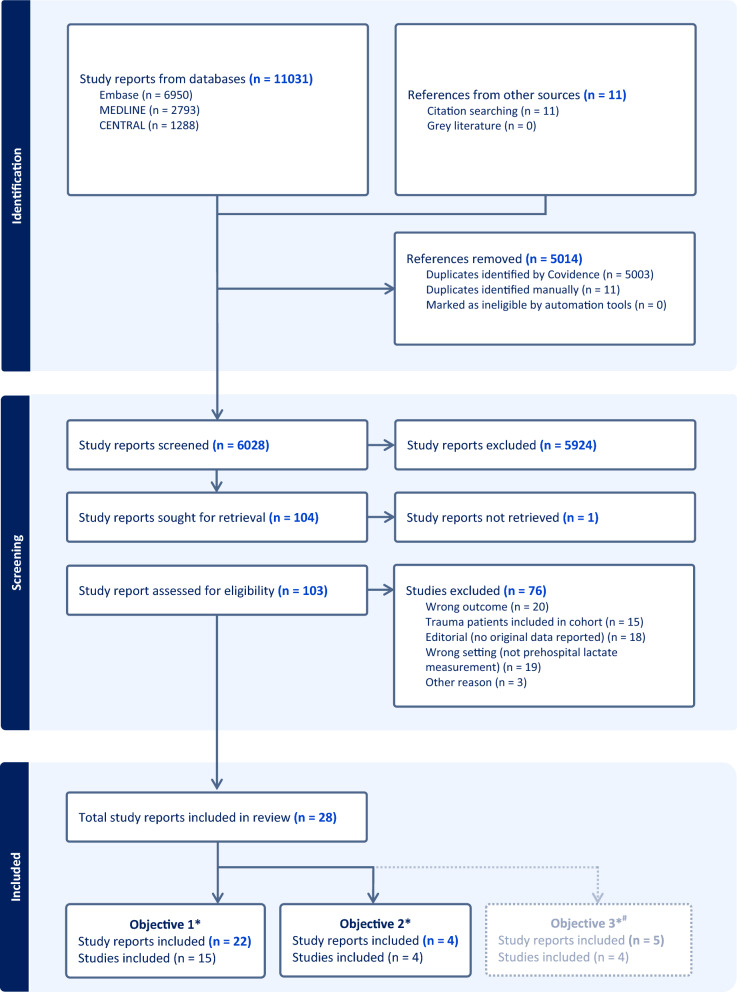
Flow chart of the literature search and the study selection process. *Numbers do not add up, as three studies were included in two of the sub-studies each. #Objective 3’s data are reported in the Supplementary Files

The search strategy was developed using a comprehensive list of keywords with assistance from an information specialist. We used a PICO-style approach using the population and the intervention [16]. The three final search strategies are shown in the Supplementary Files, Figs. 1–3.

### Selection process

The systematic review management software Covidence (Melbourne, Australia) was used during the selection process.

LHW and HBM conducted the first part of the selection process by independently screening the headings and abstracts. Afterward, they independently made the final decision of inclusion by reading the full texts. If the two researchers did not agree on study inclusion, they reached an agreement through discussion. If necessary, ACB was consulted and made the final decision.

The researchers were not blinded to any information about the reports (authors, publishing journal, etc.) during the selection process.

### Data collection process

LHW and HBM independently extracted the data from the included studies in Objective 1, using an a-priori-developed electronic data extraction form in Excel (Microsoft, Redmond, WA). LHW extracted the data in Objective 2. Afterward, ACB verified the extracted data. Disagreements were resolved through discussion between LHW and ACB.

If more than one report covered the same study population, study outcomes were extracted from the most comprehensive report. The secondary study reports were examined to extract additional information.

### Data items

Data on study outcomes were extracted according to the three objectives. We also sought information on the study setting, the number of study participants, demographic information, methodology, intervention details, financial support, etc. A list of all predefined variables extracted from the study reports is presented in the Supplementary Files, Sect. 1. In Objective 1, the first prehospital blood lactate measurement was selected in case of multiple lactate measurements in the prehospital environment.

### Study risk of bias assessment

The risk of bias (RoB) was assessed in all studies included in Objectives 1 + 2 using three preselected ‘Risk of bias’ tools. We originally planned to assess the RoB at the study level. Instead, the assessments were done at the outcome level, as several of the included studies did not have our outcome of interest (the prehospital lactate level’s correlation to the risk of poor outcomes or changes in early patient care) as their primary outcome.

The cohort studies in Objective 1 were assessed using a modified version of the QUIPS (Quality In Prognosis Studies) tool [17, 18] as recommended by the Cochrane Prognosis Methods Group [19]. Our modified QUIPS tool is shown in Supplementary Files, Fig. 4. We did not attempt to assign an overall score of the RoB. LHW and HBM did the assessments independently in Objective 1. ACB made the final decision if a disagreement between the two reviewers was not resolved by discussion.

In Objective 2, the cohort studies were assessed using the ‘Newcastle–Ottawa scale (NOS) for assessing the quality of nonrandomized studies’ modified to our primary review questions [20]. Details are described in the Supplementary Files, Sect. 2. The randomized controlled trial included in Objective 2 was assessed using the revised Cochrane ‘Risk of bias’ tool for randomized trials (RoB 2) [21].

### Synthesis methods

#### Objective 1

Instead of a meta-analysis, we planned to present the studies’ outcomes separately. We prespecified that if enough studies were included (> 4 (randomized) controlled trials or > 7 cohort studies), the studies’ outcomes would be ordered in tables according to a higher/lower risk of bias assessment. This was planned to reveal possible differences in outcomes related to the studies’ risks of bias.

The short-term mortality risks reported in the studies would be pooled to provide a total estimate of the mortality risk if two or more studies had comparable patient groups and methods for lactate measurements. We expected that the patient populations in the included cohort studies would consist of sepsis or unselected medical patients based on non-structured preliminary literature searches. If some of the included studies were restricted to specific diagnoses (for example, cardiac arrest patients, pediatric patients, etc.), these studies’ outcomes were not pooled with sepsis or non-differentiated patients.

If a study presented multiple effect measures of the same outcome, we were primarily interested in the risk ratio presented as the odds ratio. If a study did not present its results in relative risk measures, we reported the study’s lactate levels measured in the groups compared in the study. Data imputation was not allowed in our study as we did not expect the data reporting to be detailed enough to make valid changes in the data. Thus, the effect measures presented in the studies were not converted into other effect measures. Instead, the authors of a study report were asked for additional information in case of missing or unclear data presentation.

We planned to compile the results of the studies on mortality at different lactate levels. The included cohort studies were expected to divide the lactate values into intervals of low, intermediate, and high values. We planned to use the cut-off levels < 2 mmol/l, between 2 and 4 mmol/l, and > 4 mmol/l or the cut-off levels most often reported in the included studies [22, 23]. If the included cohort studies presented these data, a pooled estimation of the risk ratio of short-term mortality’s correlation to the continuous blood lactate values was also planned.

No pooled analysis was planned for outcomes other than mortality due to the anticipated few studies reporting these outcomes. Additionally, no subgroup analysis or sensitivity analysis was planned. Instead, the clinical and methodological variabilities of the studies were narratively evaluated.

#### Objective 2

We planned to present the outcomes from these studies as a narrative summary. More studies than expected were included in the Objective 2. Consequently, study outcomes were presented in a table instead to provide an overview.

Due to the anticipated few included studies, we did not plan any data synthesis, subgroup analysis, or sensitivity analysis for this objective. We discussed the studies’ heterogeneities in the text.

### Reporting bias assessment

The presence of outcome reporting bias was evaluated by searching for published study protocols of the included studies and comparing each protocol to the respective study's report(s). If a published study protocol could not be retrieved, we compared the outcomes reported in the method and results sections.

We did not plan any analysis of the risk of publication bias.

## Results

### Study selection

The literature search yielded 11,031 study reports with 5014 duplicates. The backward and forward citation search yielded an additional eleven study reports. A total of 6028 study reports were screened by titles and abstracts, and 104 study reports were assessed for eligibility by reading full texts. One study report (a conference abstract) had to be excluded from the full-text screening due to missing information and no response from the study report’s author. We excluded 15 study reports because data on the non-trauma patients were not separated from the trauma patients [1, 11, 23–35]. A total of 28 study reports were included in the review. See Fig. 1 for details.

### Study characteristics and outcomes

#### Objective 1: The prognostic value of prehospital lactate

We included 15 studies with a total of 7456 patients [12, 36–49]. They were all cohort studies and included between 83 and 1744 patients. We excluded two studies due to overlapping patients after contact with the first authors and used their study reports as supplemental information [50, 51].

The lactate levels were blinded to all treating personnel in one study, where the prehospital lactate levels were analyzed using stationary in-hospital analyzers [36]. The remaining 14 studies used various point-of-care equipment and analyzed the lactate levels as a part of the prehospital treatment provided to the patients. Eleven studies were prospective, and four were retrospective cohort studies. Eleven studies were conducted in Europe, three in the United States, and one in Australia. Four studies investigated patients with suspected sepsis or septic shock. Three studies examined patients with out-of-hospital cardiac arrest (OHCA). All studies were conducted in adult patients ≥ 18 years old, and the inclusion periods ranged from 2010 to 2021. Eleven studies investigated the short-term mortality risks (≤ 30-day or in-hospital mortality). None of the included studies investigated adverse events related to the lactate measurements.

The studies’ details and outcomes are presented in Table 1. The studies’ RoB assessments are displayed in Table 2. Both tables were ordered according to the studies’ RoB, with the study with the lowest RoB at the top of both tables. Most studies had moderate or high RoB in the confounding domain. No study had low RoB in all six domains.

**Table 1:** Objective 1. Cohort studies investigating the correlation between prehospital measured blood lactate levels and patients’ prognoses

First author, year, References	Study design	Country	Study period	Sample size (N)	Patients	Age, years	Lactate levels (mmol/L), sample site, Device	Outcomes	Main findings
Ivic 2021 [36]	Prospective **Blinded**	Sweden & Finland	2015–2017	414	Non-specific complaints	82 (median)	≤ 2.2 vs. ≥ 2.3 Venous, In-hospital equipment	30-day mortality	The median lactate level was 1.7 mmol/L (IQR 1.3–2.3) in survivors and 1.8 mmol/L (IQR 1.7–3.3) in non-survivors 10/288 patients (3.5%) died in the group with lactate ≤ 2.2 mmol/L vs. 7/109 patients (6.4%) in the group with lactate ≥ 2.3 mmol/L. p = 0.09
del Pozo Vegas 2023 [37]	Prospective	Spain	2019–2020	1744	Acute cardiovascular disease	62 (pt. < 75 years) 84 (pt. ≥ 75 years) (median)	Continuous, ?, Epoc (Siemens)	365-day mortality	The mean lactate level was 1.99 mmol/L (SD 1.35) in survi-vors vs. 4.63 mmol/L (SD 3.79) in non-survivors. p < 0.001. Multivariate Cox regression: HR 1.25 (95% CI 1.20–1.30) p < 0.001
Jouffroy 2020 [38]	Prospective	France	2017–2019	177	Septic shock	70 (mean)	Continuous & < 4.0 vs. ≥ 4.0, Venous, StatStrip (Nova Biomed.)	30-days mortality	The mean lactate level was 5.9 mmol/L (SD 3.5) in survivors and 7.1 mmol/L (SD 4.0) in non-survivors. P < 0.001 The OR for 30-day mortality (adjusted for confounders) = 2.95 (95% CI 1.14–9.18) in patients with lactate levels ≥ 4.0 mmol/L. P < 0.001
Jouffroy 2022 (Sepsis) [39]	Retrospective	France	2016–2020	406	Septic shock	69 (mean)	Continuous, ?, StatStrip (Nova Biomed.)	28-days mortality	The mean lactate level was 5.8 mmol/L (SD 3.5) in survivors vs. 6.9 mmol/L (SD 4.0) in non-survivors. p = 0.059
Martín-Rodríguez 2020 [40]	Prospective	Spain	2018–2019	492	Acute cardiovascular disease	72 (median)	Continuous, Venous, Accutrend Plus (Roche)	48-h mortality	The median lactate level was 2.8 mmol/L (IQR 2.1–3.8) in survivors vs. 6.2 mmol/L (IQR 4.5–7.9) in non-survivors p < 0.001
Martín-Rodríguez 2021 [41]	Prospective	Spain	2018–2019	221	Acute poisoning	47 (median)	≤ 3.9 vs. ≥ 4.0, Venous, Accutrend Plus (Roche)	Serious adverse events including 30-day mortality	35/183 (19.1%) had a lactate level ≥ 4.0 mmol/L in patients without serious adverse events vs. 21/38 (55.3%) in patients with serious adverse events. Only 9/221 (4.1%) died
Tobias 2014 [42]	Prospective Convenience	USA	2010–2011	673	Unselected	62 (grp. 1) 64 (grp. 2) (mean)	< 2.0 (grp. 1) vs ≥ 2.0 (grp. 2), Venous, Lactate Pro (FACT)	In-hospital mortality	5/366 patients (1.4%) died in the group with lactate < 2.0 mmol/L vs. 16/307 patients (5.2%) in the group with lactate ≥ 2.0 mmol/L. p < 0.01 Multivariate logistic regression: OR = 3.57 (95% CI 1.10–11.60) with a lactate level ≥ 2.0 mmol/L. p = 0.03
Corral Torres 2020 [43]	Prospective	Spain	2012–2017	1552	OHCA*	64.8 (mean)	Continuous, Venous, epoc (Epocal Inc.)	Good neurological outcome (CPC I-II) after 30 days	The mean lactate level was 7.11 mmol/L (SD 17.63) in patients with CPC I-II vs. 7.26 mmol/L (SD 6.63) in patients with CPC III-V including non-survivors OR of CPC III-V = 1.002 (0.989–1.014), p = 0.804
Villanueva 2021 [44]	Prospective	Spain	2018–2019	638	Dyspnoea (medical)	79 (median)	Continuous, Venous, Accutrend Plus (Roche)	7-day mortality	OR of 7-day mortality was 9.11 (95% CI 5.48–15.13) with a lactate level ≥ 4.1 mmol/L. p < 0.001
Swan 2019 [45]	Retrospective	Australia	2014–2015	253	Unselected	75 (median)	Continuous, Venous or capillary, Lactate Pro 2 (ARKRAY)	In-hospital mortality ICU admission	The median lactate level was 2.4 mmol/L (IQR 1.5–3.6) in survivors vs. 3.5 mmol/L (IQR 2.75–5.85) in non-survivors p = 0.053 The median lactate level was 2.4 mmol/L (IQR 1.5–3.6) in non-ICU patients vs. 3.2 mmol/L (IQR 2.4–5.7) in ICU patients. p = 0.008
Boland 2016 [12]	Prospective Convenience	USA	2011–2013	112	Sepsis	74 (mean)	< 4.0 vs. ≥ 4.0, Capillary, Lactate Pro (KDK Corp)	In-hospital mortality ICU admission	In-hospital mortality was 1% (1/99) in patients with lactate levels < 4.0 mmol/L vs. 18% (2/13) in patients with lactate levels ≥ 4.0 mmol/L. P = 0.04 The ICU admission rate was 15% (15/99) in patients with lactate levels < 4.0 mmol/L vs. 23% (3/13) in patients with lactate levels ≥ 4.0 mmol/L. P = 0.44
Gruebl 2021 [46]	Retrospective	Germany	2015–2016	98	OHCA*	69 (mean)	Continuous, Venous or arterial, epoc (Epocal Inc.)	In-hospital mortality	The mean lactate level was 6.1 mmol/L in survivors vs. 8.5 mmol/L in non-survivors. p = 0.131
Vujanovic** 2023 [47]	Prospective	Slovenia	2020–2021	83	OHCA*	67 (median)	Continuous, Capillary, BM-Lactate Cobas (Roche)	7-day mortality	The median first measured lactate level was 1.85 mmol/L (IQR 0.8–13.58) in survivors (N = 6) vs. 5.9 mmol/L (IQR 2.70–10.9) in non-survivors (N = 70) T-test comparing the mean lactate levels in the two groups: p = 0.546
Jouffroy 2022 (COVID) [48]	Retrospective	France	2020	410	COVID-19	64 (mean)	Continuous, ?, StatStrip (Nova Biomed.)	30-day mortality ICU admission	Multivariate logistic regression: OR = 0.87 (95% CI 0.68–1.08). p = 0.246 Multivariate logistic regression: OR = 1.14 (95% CI 0.89–1.43). p = 0.29
Shiuh*** 2012 [49]	Prospective	USA	2011–2012	183	Sepsis	?	2.5–3.9 vs. ≥ 4.0, Venous, ?	ICU admission	The ICU admission rate was 23% (22/97 patients) in the group with lactate levels 2.5–3.9 mmol/L vs. 50% (43/86 patients) with lactate levels ≥ 4.0 mmol/L

The table is divided into studies with blinded lactate values at the top and studies with unblended lactate values at the bottom. The studies are ordered according to their risk of bias, with the study with the lowest risk displayed first in the table. The primary outcome, short-term mortality, is displayed along with the other outcomes reported from the included studies. Unless otherwise stated, all data was extracted from the primary study report of each study. Study periods are rounded off to years

*OHCA = out-of-hospital cardiac arrest

**The median lactate level data was obtained by communicating with the study report's first author

***The study report was a conference abstract, and we could not retrieve additional data

**Table 2:**
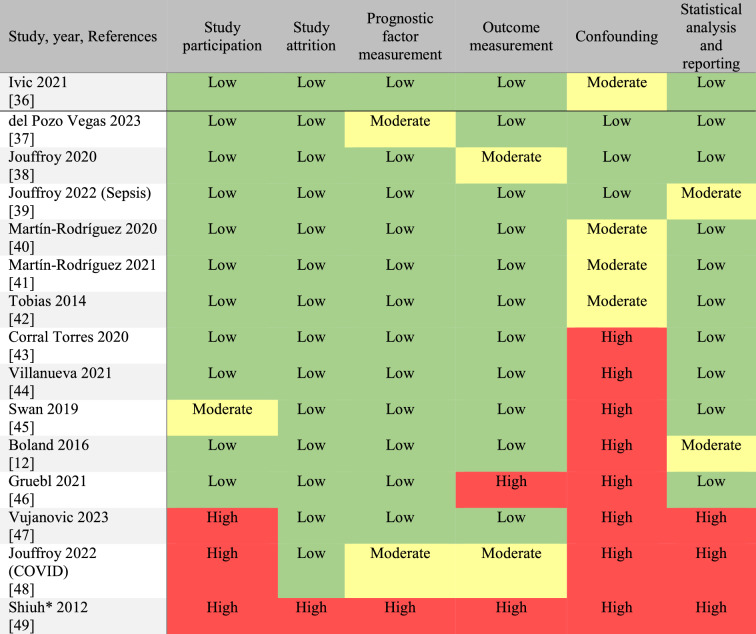
Risk of bias assessments in the studies included in Objective 1

The QUIPS (Quality In Prognosis Studies) tool was used for the risk of bias assessments. Studies with blinded lactate results are displayed above the black line, and studies without blinded lactate results are placed below the black line. The studies were ordered according to their risk of bias, with the study with the lowest risk of bias displayed at the top of the table. If they had equal risks of bias, the studies were listed alphabetically

*The study report was a conference abstract, and we could not retrieve additional information

#### Sepsis patients

Three studies presented short-term mortality data in 695 patients, of which 289 were assessed prehospital with suspected sepsis/septic shock, and 406 patients were a mix of hospital-verified and prehospital suspicion of sepsis/septic shock [12, 38, 39]. The studies’ data were not fit for pooled analysis. Two studies showed significantly higher risks of in-hospital mortality in sepsis patients with elevated lactate levels ≥ 4.0 mmol/L [12, 38]. The largest study with 406 patients did not find clear evidence of a difference in lactate levels in survivors and non-survivors (p = 0.059) [39].

Two of the included studies were conducted by the same investigators with overlapping periods [38, 39]. Thus, we expected these two cohorts to include some of the same patients. Communication with the authors did not confirm this suspicion.

#### Patients with OHCA

Two studies investigated short-term mortality, and one study reported good vs. poor neurological outcomes (including death) at 30 days in a total of 1733 patients with OHCA.[43, 46, 47] None of the studies found evidence of a difference in the mean lactate levels in survivors/good neurological outcomes versus non-survivors/poor neurological outcomes. The data reported from the studies were not fit for pooled analysis.

#### Patients with various diseases

The remaining six studies reporting short-term mortality risks did not have comparable patient populations [36, 40, 42, 44, 45, 48]. Two studies investigated unselected non-trauma patients, and one study each included patients with acute cardiovascular disease, dyspnea, non-specific complaints, and COVID-19, respectively. The studies’ data were not pooled. Two of the studies reported clear evidence of a correlation between elevated lactate levels and higher risks of short-term mortality in patients with acute cardiovascular disease and dyspnea, respectively [40, 44]. Three studies reported moderate evidence of this correlation in patients with non-specific complaints and unselected non-trauma patients [36, 42, 45]. The last study did not find evidence of any correlation between the prehospital lactate level and short-term mortality risk in COVID-19 patients [48]. This study had the highest risk of bias compared to the other five studies.

#### Objective 2: The prehospital lactate levels’ impact on changes/modifications in early patient care

We included four studies: two retrospective cohort studies, one prospective cohort study, and one randomized controlled trial [46, 52–54]. One study investigated the prehospital lactate level as a single test [53]. The three remaining studies investigated a panel of tests, including a lactate level, used in the treatment of prehospital patients [46, 52, 54]. Three of the studies showed changes in prehospital sepsis care in patients with prehospital lactate measurements [52–54]. One study reported additional prehospital administration of antibiotics in 42 out of 68 patients with suspected sepsis because their prehospital lactate level was > 4.0 mmol/L. This treatment was part of a treatment flow chart in this study’s setting [52]. The study by Younger et al. did not report specific changes in the care for sepsis patients, and only a few sepsis patients were included in the study by Zwisler et al. The data are presented in Table 3.

**Table 3:** Studies investigating the impact of prehospital lactate levels on changes/modifications in early patient care

First author, Year, Reference	Study design	Setting	Patients	N	Measurement (Device)	Change in prehospital care
Collopy, 2022 [52]	Retrospective cohort	Air/ground Emergency Service + interfacility transport, USA	All	632	A panel of tests including a lactate level. (Epoc)	Antibiotics were administrated during transport in 42/68 patients (61.8%) with sepsis/ septic shock because of a lactate level > 4.0 mmol/L Patient care was altered in 10/28 patients (35.7%) with heart failure including administrating dobutamine in case of a lactate level > 4.0 mmol/L
Gruebl, 2021 [46]	Retrospective cohort	Prehospital Emergency Service, Germany	Out-of-hospital cardiac arrest	263	A panel of tests including a lactate level. (Epoc)	Intravenous sodium bicarbonate was administered in 61/98 patients (62%) with a point-of-care blood test vs. in 23/165 patients (14%) with no test. p < 0.001
Younger, 2014, [53]	Prospective cohort	Prehospital Emergency Service, England	Sepsis	109	Lactate level (StatStrip)	The decision to treat the patient for sepsis was affected in 54/90 cases (60%) due to the lactate test result
Zwisler, 2019, [54]	Randomized controlled trial	Prehospital Emergency Service, Denmark	Critically ill (Glasgow Coma Score < 13)	222	A panel of tests including a lactate level. (ABL90)	The test results led to 159 interventions in 71/102 patients (69.6%) in the group randomized to a blood test. Sepsis treatment including prehospital antibiotics was done in 3/102 patients (2.9%)

Studies are listed alphabetically by author name. Unless otherwise stated, all data was extracted from the primary study report of each study

Our systematic review did not find any studies reporting changes in the fluid administration, blood transfusion(s), oxygen supply, or triage, nor did we find studies investigating possible changes in early *in-hospital* patient care based on prehospital lactate measurements.

We assessed all four studies as having high risks of bias (see Table 4).

**Table 4:**
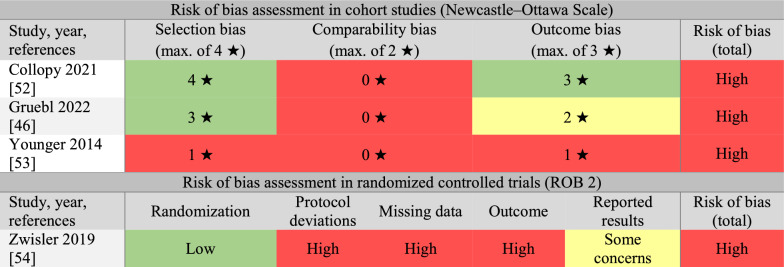
Risk of bias assessments in the studies included in Objective 2

The Newcastle–Ottawa Scale was used to assess the risks of biases in cohort studies. The revised Cochrane “Risk of bias” tool for randomized trials (ROB 2) was used to assess the risk of bias in the randomized controlled trial. The studies were listed in alphabetical order

### Outcome reporting bias

#### Objective 1

We found only one published study protocol among the studies included in Objective 1 [55]. The study's results were later reported in two study reports [38, 50]. The study protocol was not complied with in all aspects, but the analysis and reporting of the primary outcome (30-day mortality) remained unaltered.

The method and results sections were compared in all the included study reports in Objective 1. No selective reporting was observed of the question addressed in this objective. One study’s results were reported as a conference abstract, and the risk of reporting bias was impossible to evaluate in this study [49].

#### Objective 2

We did not detect any selective reporting of results in this objective. However, only two study reports included information about their planned analyses in their method sections. It was impossible to evaluate the remaining two studies’ risks of reporting bias.

## Discussion

Evidence from the studies included in our systematic review demonstrates that elevated prehospital lactate levels increased the risk of poor outcomes in most non-trauma patients but not in patients with OHCA. Limited data show changes in early prehospital care in sepsis patients, such as prehospital administration of antibiotics based on their prehospital measured lactate levels.

### Cut-off lactate levels

A lactate cut-off level is relevant to efficient risk assessment in a clinical setting. A proper cut-off level may be particularly beneficial in patients at risk but without clear signs of deterioration. We expected most studies to use cut-off levels and designed this systematic review on these grounds. Surprisingly, only one-third of the included studies did so, with the majority using a cut-off of around 4 mmol/L.

Previous *in-hospital* studies mainly included a cut-off level of around 2.0–2.5 mmol/L, and the authors argue that an even lower threshold for serial in-hospital lactate measurements should be considered [2]. A prehospital cut-off lactate level of around 2.0 mmol/L to deem a risk of severe illness is likely to have a high sensitivity but will lead to excessive over-triage. Just two out of 13 sepsis patients with elevated prehospital lactate levels had a sustained lactate level ≥ 4.0 mmol/L at admission [12].

A prehospital lactate level cut-point around 4.0 mmol/L could be considered reasonable as it is often measured very early in the course of the disease and before initial treatment. However, this systematic review did not aim to evaluate the cut-off levels used in the included studies.

### Objective 1: The prognostic value of prehospital lactate

Most of the included studies found evidence of a correlation between elevated prehospital lactate levels and poor outcomes. These results are in line with previous findings in in-hospital patients and prehospital trauma patients [2, 3, 9]. The correlation persisted in patients with various illnesses such as sepsis, cardiovascular diseases, dyspneic patients, and in unselected prehospital patients. There was a tendency to find more significant results in the studies with the lowest RoB. Thus, our confidence increased that a correlation between elevated prehospital lactate levels and poor outcomes persisted in non-trauma patients. The correlation was present in both short-term and long-term mortality and admission to the ICU.

One of the included studies blinded the patients’ lactate levels to all participants [36]. They did not find clear evidence of a correlation between lactate levels and 30-day mortality in patients with non-specific complaints (p = 0.09). The study used a low cut-off lactate level of 2.2 mmol/L, which may be the reason for no apparent difference in mortality risk between the two groups.

#### Sepsis patients

The Sepsis-3 Consensus Definition advocates prompt lactate measurements in suspected sepsis patients [8]. Two of three prehospital studies included in this review support this recommendation [12, 38]. One study indicated that prehospital serial lactate measurements could be superior to just one initial lactate measurement in prognostication [39]. Monitoring prehospital lactate clearance might be beneficial, particularly in long transfer times.

#### Patients with OHCA

The three studies on OHCA patients included in this systematic review did not find evidence of a correlation between elevated prehospital lactate levels and poor outcomes. This differs from studies examining the in-hospital lactate levels in OHCA patients with and without extracorporeal circulation [56, 57]. An explanation could be a difference in OHCA study patients’ characteristics. OHCA patients with and without transportation to the hospital are not comparable.

The difference between the studies' results may be due to the timing of the lactate measurement. The lactate levels are expected to be elevated in all OHCA patients during a period with low flow. However, studies with in-hospital cardiac arrest (IHCA) and assumed short response times have also found clear evidence of a correlation between elevated lactate levels and poor outcomes [58]. Still, patients with IHCA and patients with OHCA are not comparable in comorbidities or the reasons for cardiac arrests [59].

The increased use of prehospital advanced treatments such as extracorporeal cardiopulmonal resuscitation in OHCA calls for prehospital prognostic tools [60]. The MIRACLE-2 score has proven reliable in OHCA prognostication in patients with cardiac origin [61]. The score includes the initial pH level obtained at hospital arrival. The prehospital lactate level may assist in prehospital risk stratification before initiating advanced prehospital treatments, possibly combined with other known risk factors in a prehospital prognostic tool.

### Objective 2

We found sparse evidence on the effect of prehospital lactate levels on changes/modifications in early patient care. Most changes were documented in sepsis patients. Antibiotic treatments were administered in 61.8% of septic patients during transport due to the lactate measurements (as noted in a flow chart in the study report) [52]. The only study investigating the lactate level as a sole measurement did not specify what changes were made due to the measurement [53]. The study by Zwisler et al., included in our review, reported many interventions made due to the prehospital blood analyses in patients with impaired consciousness. Unfortunately, as the lactate measurement was a part of a panel of blood tests, it is not possible to make probable correlations between the interventions and the lactate measurement alone.

Another systematic review from 2017 included eight studies to evaluate the effect of lactate measurements in sepsis patients at presentation to health care [3]. One study included in that review was from the prehospital environment and included inter-hospital aeromedical transfers, and the rest of the included studies were investigating in-hospital lactate measurements. That systematic review showed that lactate measurements reduced the time to intravenous fluid and antibiotic administration in most of the included studies. The only prehospital study included in that review did not find a difference in the number of intubations or insertion of central venous lines between patients with and without lactate measurements, but the study was probably underpowered.

Three of the four studies in Objective 2 did not report patients’ outcomes [52–54]. The fourth study did not specify the changes made due to lactate measurements alone [46]. Consequently, the impact of the treatment changes made due to the lactate measurements (as a sole analysis or as a part of a blood test panel) cannot be assessed.

### Future research

This study’s findings indicate that prehospital lactate levels can be used in the early prognostic assessment of most non-trauma patients. This aligns with the evidence on the in-hospital use of lactate measurements.

Surprisingly, prehospital lactate levels did not seem to differ between survivors and non-survivors in patients with OHCA, contrary to lactate levels measured in-hospital. More studies with larger cohorts targeting this population are needed to investigate this correlation, as two out of three studies included in our systematic review had few included patients. The studies’ risks of survivor bias also need to be addressed to increase their findings’ generalizability to other prehospital settings.

Most studies on prehospital lactate measurements focus on the patients’ prognoses. Little evidence exists on the changes in early patient treatment. Future research should focus on whether prehospital lactate measurements change how we treat the patients and if these changes improve patients’ outcomes. These studies should ideally be randomized controlled trials with a lactate measurement as the intervention compared to no lactate measurement to minimize the risk of bias.

### Limitations

This systematic review included studies with substantial heterogeneity. In addition, several studies were small and probably underpowered, especially in Objective 2. None of the included studies had a low risk of bias, and all studies in Objectives 2 and 3 had a high risk of bias. This induces low quality of the body of evidence, particularly in Objectives 2 and 3.

Surprisingly, most studies in Objective 1 (6/9, 67%) reported continuous lactate levels as means instead of medians. This error can introduce crucial bias to the results. This is particularly important in studies with few patients, where one or a few outliers can substantially change the mean measurement. Thus, our confidence in the evidence presented in these studies decreased.

Many large studies had to be excluded because of our predefined decision to exclude studies with both non-trauma and trauma patients unless the results were separated between groups. This decision yielded less power but potentially more precision to our systematic review. On the contrary, our decision to include in-hospital mortality as short-term mortality may have decreased the accuracy of Objective 1’s results. If the patients died after discharge but within 30 days, they were not recorded as deceased in the studies using the outcome of in-hospital mortality. Additionally, in-hospital mortality may contain patients with a survival of more than 30 days. However, as many studies use in-hospital mortality as their outcome, we decided a priori to include this outcome in our systematic review to include more potential studies.

We did not plan to assess the risk of publication bias. However, the risk of publication bias was expected to be high, as studies with down to 20 patients could be included in our review. Small studies with no difference between study groups’ outcomes are less likely to be published than small studies with significant findings.

## Conclusions

Elevated prehospital lactate levels were correlated to increased short-term mortality in most acute non-trauma patients but not in patients experiencing OHCA. Few studies suggest that prehospital lactate measurements may impact interventions in septic patients. The included studies were heterogeneous, and most had high risks of bias. Further studies are needed to investigate the impact of prehospital lactate measurements on patient care and whether this affects patients’ outcomes.

## Supplementary Information

Supplementary Material 1

## Data Availability

All additional data are available upon reasonable request by contacting the corresponding author.

## Abbreviations

ICU: Intensive care unit
IHCA: In-hospital cardiac arrest
NOS: Newcastle–Ottawa scale
OHCA: Out-of-hospital cardiac arrest
QUIPS: Quality In Prognosis Studies
RoB: Risk of bias
