# Supplementary material for: The use and impact of prehospital blood lactate measurements in acute non-traumatic patients: a systematic review
Source: Scand J Trauma Resusc Emerg Med. 2024 Dec 26;32:137. doi: 10.1186/s13049-024-01310-1 (PMC11670429; doi:10.1186/s13049-024-01310-1)
Supplement: Supplementary file 1 — Supplementary Material 1 [file 13049_2024_1310_MOESM1_ESM.docx]

**Supplementary Files**

**Figure 1:** Literature search in Medline (Ovid)

**Ovid MEDLINE(R) and Epub Ahead of Print, In-Process, In-Data-Review & Other Non-Indexed Citations and Daily <1946 to August 26, 2023>**

| **#** | **Searches** | **Results** | **Type** |  |  |  |
| --- | --- | --- | --- | --- | --- | --- |
|  | | | | | | |
| 1 | Emergency Medical Services/ | 48704 | Advanced |  |  |  |
| 2 | Air Ambulances/ or Ambulances/ | 9958 | Advanced |  |  |  |
| 3 | point-of-care testing/ or rapid diagnostic tests/ or rapid on-site evaluation/ | 4155 | Advanced |  |  |  |
| 4 | (prehospital or pre-hospital or out-of-hospital).mp. [mp=title, book title, abstract, original title, name of substance word, subject heading word, floating sub-heading word, keyword heading word, organism supplementary concept word, protocol supplementary concept word, rare disease supplementary concept word, unique identifier, synonyms, population supplementary concept word, anatomy supplementary concept word] | 35412 | Advanced |  |  |  |
| 5 | ((emergency adj2 servic*) or (accident adj2 servic*)).mp. [mp=title, book title, abstract, original title, name of substance word, subject heading word, floating sub-heading word, keyword heading word, organism supplementary concept word, protocol supplementary concept word, rare disease supplementary concept word, unique identifier, synonyms, population supplementary concept word, anatomy supplementary concept word] | 145382 | Advanced |  |  |  |
| 6 | ((emergency adj2 care) or (acute adj2 care) or (prehospital adj2 care) or (pre-hospital adj2 care)).mp. [mp=title, book title, abstract, original title, name of substance word, subject heading word, floating sub-heading word, keyword heading word, organism supplementary concept word, protocol supplementary concept word, rare disease supplementary concept word, unique identifier, synonyms, population supplementary concept word, anatomy supplementary concept word] | 58859 | Advanced |  |  |  |
| 7 | (ambulance* or emergency car* or emergency vehicle* or emergency mobile unit*).mp. [mp=title, book title, abstract, original title, name of substance word, subject heading word, floating sub-heading word, keyword heading word, organism supplementary concept word, protocol supplementary concept word, rare disease supplementary concept word, unique identifier, synonyms, population supplementary concept word, anatomy supplementary concept word] | 31768 | Advanced |  |  |  |
| 8 | (point of care or point-of-care).mp. [mp=title, book title, abstract, original title, name of substance word, subject heading word, floating sub-heading word, keyword heading word, organism supplementary concept word, protocol supplementary concept word, rare disease supplementary concept word, unique identifier, synonyms, population supplementary concept word, anatomy supplementary concept word] | 41069 | Advanced |  |  |  |
| 9 | 1 or 2 or 3 or 4 or 5 or 6 or 7 or 8 | 251611 | Advanced |  |  |  |
| 10 | exp lactic acid/ | 48427 | Advanced |  |  |  |
| 11 | (lactat* or lactic acid* or L-lactic acid* lactic).mp. [mp=title, book title, abstract, original title, name of substance word, subject heading word, floating sub-heading word, keyword heading word, organism supplementary concept word, protocol supplementary concept word, rare disease supplementary concept word, unique identifier, synonyms, population supplementary concept word, anatomy supplementary concept word] | 298043 | Advanced |  |  |  |
| 12 | 10 or 11 | 299782 | Advanced |  |  |  |
| 13 | 9 and 12 | 2105 | Advanced |  |  |  |
| 14 | limit 13 to human | 1665 | Advanced |  |  |  |

**Figure 2:** Literature search in Embase (Ovid).

**Embase Classic+Embase <1947 to 2023 August 26>**

| **#** | **Searches** | **Results** | **Type** |  |  |  |  |  |
| --- | --- | --- | --- | --- | --- | --- | --- | --- |
|  | | | |  |  |  |  |  |
| 1 | emergency health service/ | 118417 | Advanced |  |  | | |  |
| 2 | (prehospital or pre-hospital or out-of-hospital).mp. [mp=title, abstract, heading word, drug trade name,original title, device manufacturer, drug manufacturer, device trade name, keyword heading word, floating subheading word, candidate term word] | 54653 | Advanced |  |  | | |  |
| 3 | ((emergency adj2 servic*) or (accident adj2 servic*)).mp. [mp=title, abstract, heading word, drug trade name, original title, device manufacturer, drug manufacturer, device trade name, keyword heading word, floating subheading word, candidate term word] | 139763 | Advanced |  |  | | |  |
| 4 | exp emergency care/ | 60975 | Advanced |  |  | | |  |
| 5 | ((emergency adj2 care) or (acute adj2 care) or (prehospital adj2 care) or (pre-hospital adj2 care)).mp. [mp=title, abstract, heading word, drug trade name, original title, device manufacturer, drug manufacturer, device trade name, keyword heading word, floating subheading word, candidate term word] | 116755 | Advanced |  |  | | |  |
| 6 | exp ambulance transportation/ or exp ambulance/ | 18522 | Advanced |  |  | | |  |
| 7 | (ambulance* or emergency car* or emergency vehicle* or emergency mobile unit*).mp. [mp=title,  abstract, heading word, drug trade name, original title, device manufacturer, drug manufacturer, device trade name, keyword heading word, floating subheading word, candidate term word] | 94559 | Advanced |  |  | | |  |
| 8 | exp "point of care testing"/ | 21680 | Advanced |  |  | | |  |
| 9 | (point of care or point-of-care).mp. [mp=title, abstract, heading word, drug trade name, original title, device manufacturer, drug manufacturer, device trade name, keyword heading word, floating  subheading word, candidate term word] | 54898 | Advanced |  |  | | |  |
| 10 | 1 or 2 or 3 or 4 or 5 or 6 or 7 or 8 or 9 | 335389 | Advanced |  |  | | |  |
| 11 | exp lactic acid/ | 106602 | Advanced |  |  | | |  |
| 12 | (lactat* or lactic acid* or L-lactic acid* lactic).mp. [mp=title, abstract, heading word, drug trade name, original title, device manufacturer, drug manufacturer, device trade name, keyword heading word, floating subheading word, candidate term word] | 454235 | Advanced |  |  | | |  |
| 13 | 11 or 12 | 454235 | Advanced |  |  | | |  |
| 14 | 10 and 13 | 4942 | Advanced |  |  | | |  |
| 15 | limit 14 to human | 4382 | Advanced |  |  | | |  |
|  | | |  | | |  |  |  |

**Figure 3:** Literature search in Cochrane Central Register of Controlled Trials (Wiley)


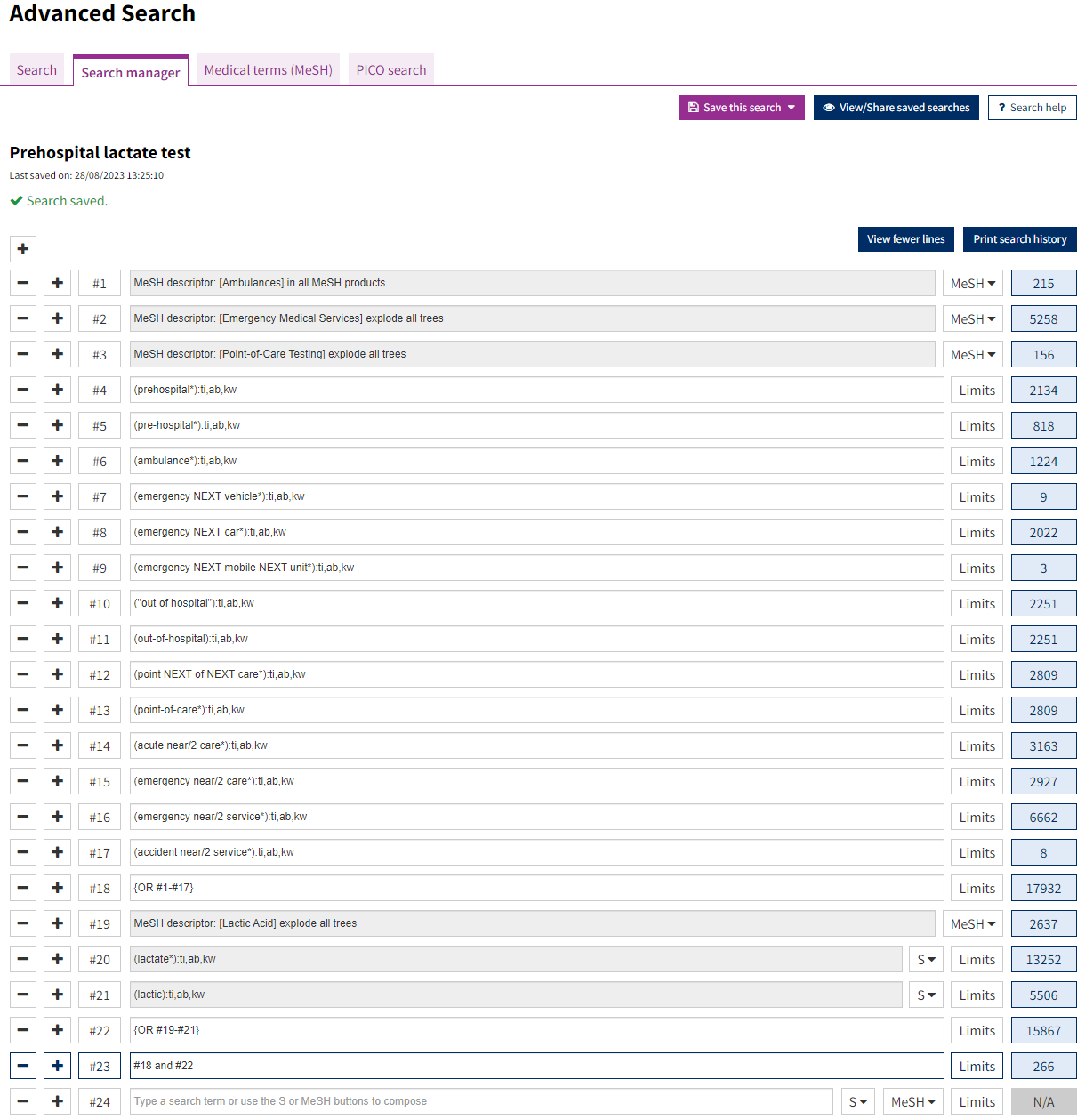


**Section 1:** A list of all variables we sought to extract from the included studies.

Objectives 1+2

Methods:

- Study design
- Blinding and sequence generation (in case of RCT)
- Total study duration

Participants:

- Total number
- Age (median or mean)
- Sex
- Setting (countries, ground-based/helicopter, urban/rural, or a combination)
- Specific illness (seizures, heart disease, medical, children, or a mixed population)
- Comorbidity variables

Interventions:

- Device name and manufacture
- Sample site (arterial, venous, and/or capillary)
- Number of analyses (one, two, or more)
- Profession of the person performing the test
- Education time needed before implementation

Outcomes:

- Objective 1: Short-term mortality. Preferably, 7-day mortality, but up to 30 days mortality was accepted if this was the shortest period reported. The mortality rate closest to 7 days was selected if two or more mortality rates were displayed. If only in-hospital mortality was stated, this would also be a valid outcome in this study.
- Objective 1: If noted in the study reports; long-term mortality (>30 days) and secondary outcomes such as morbidity rates (number of participants admitted to ICUs, length of hospital stay, length of ICU stay, need of intubation or vasopressor, duration of ventilation support, duration of vasopressor treatments etc.)
- Objective 2: Noted altered prehospital treatment based on the measured lactate level
- Objectives 1 and 2: Reported adverse events

Other:

- Financial funding
- Ethical study approval

Objective 3:

- Number of clinicians who participated in the study
- Setting (countries, ground-based/helicopter, urban/rural, or a combination)
- Specific illness (seizures, heart disease, medical, children, or a mixed population)
- Device name and manufacture
- Sample site (arterial, venous, or capillary)
- Noted clinicians’ points of view on whether and how the lactate measurement helps them in early decision-making.

**Supplementary Figure 4:**

QUIPS Risk of Bias Assessment Instrument for Prognostic Factor Studies

*Modified from:* Hayden JA, Côté P, Bombardier C. Evaluation of the Quality of Prognosis Studies in Systematic Reviews. Annals of Internal Medicine. 2006;144:427-437, with the assistance of the

QUIPS-LBP Working Group.

| **Author and year of**  **publication** |  | | | |
| --- | --- | --- | --- | --- |
| **Study identifier** |  | | | |
| **Reviewer** |  |  |  |  |
|  |  |  |  |  |
| **Biases** | **Issues to consider for judging overall rating of "Risk of bias"** | **Study Methods & Comments** | **Rating of reporting** | **Rating of "Risk of bias"** |
| Instructions to assess the risk of each potential bias: | These issues will guide your thinking and judgment about the overall risk of bias within each of the 6 domains. Some 'issues' may not be relevant to the specific study or the review research question. These issues are taken together to inform the overall judgment of potential bias for each of the 6 domains. | Provide comments or text exerpts in the white boxes below, as necessary, to facilitate the consensus process that will follow. | Click on each of the blue cells and choose from the drop down menu to rate the adequacy of reporting as yes, partial, no or unsure. | Click on the green cells; choose from the drop-down menu to rate potential risk of bias for each of the 6 domains as High, Moderate, or Low considering all relevant issues |
| **1. Study Participation** | **Goal: To judge the risk of selection bias (likelihood that relationship between *PF* and**  ***outcome* is different for participants and eligible non-participants).** |  |  |  |
| *Source of target population* | The source population or population of interest is adequately described for key characteristics (LIST). | Less important, just a brief statement of the population |  |  |
| *Method used to identify population* | The sampling frame and recruitment are adequately described, including methods to identify the sample sufficient to limit potential bias (number and type used, e.g., referral patterns in health care) |  |  |  |
| *Recruitment period* | Period of recruitment is adequately described |  |  |  |
| *Place of recruitment* | Place of recruitment (setting and geographic location) are adequately described |  |  |  |
| *Inclusion and exclusion criteria* | Inclusion and exclusion criteria are adequately described (e.g., including explicit diagnostic criteria or “zero time” description). |  |  |  |
| *Adequate study participation* | There is adequate participation in the study by eligible individuals | >70% ="yes", >50% = "partial" |  |  |
| *Baseline characteristics* | The baseline study sample (i.e., individuals entering the study) is adequately described for key characteristics (LIST). | If age OR sex is missing = no, if only comorbidity is missing = partial |  |  |
| **Summary Study participation** | **The study sample represents the population of interest on key characteristics, sufficient to limit**  **potential bias of the observed relationship between PF and outcome.** |  |  |  |
|  | | | | |
| **2. Study Attrition** | **Goal: To judge the risk of attrition bias (likelihood that relationship between *PF* and**  ***outcome* are different for completing and non-completing participants).** |  |  |  |
| *Proportion of baseline sample available*  *for analysis* | Response rate (i.e., proportion of study sample completing the study and providing outcome data) is adequate. | Loss to follow up > 10% = partial, >20% = no |  |  |
| *Attempts to collect information on*  *participants who dropped out* | Attempts to collect information on participants who dropped out of the study are described. |  |  |  |
| *Reasons and potential impact of*  *subjects lost to follow-up* | Reasons for loss to follow-up are provided. |  |  |  |
| *Outcome and prognostic factor information on those lost to follow-up* | Participants lost to follow-up are adequately described for key characteristics (LIST). | Age & sex |  |  |
|  | There are no important differences between key characteristics (LIST) and outcomes in participants who completed the study and those who did not. |  |  |  |
| **Study Attrition Summary** | **Loss to follow-up (from baseline sample to study population analyzed) is not associated with key characteristics (i.e., the study data adequately represent the sample) sufficient to limit potential bias to the observed relationship between PF and outcome.** |  |  |  |
|  | | | | |
| **3. Prognostic Factor**  **Measurement** | **Goal: To judge the risk of measurement bias related to how PF was measured (differential measurement of PF related to the level of outcome).** | PF = lactate measurement |  |  |
| *Definition of the PF* | A clear definition or description of 'PF' is provided (e.g., including dose, level, duration of exposure, and clear specification of the method of measurement). |  |  |  |

| *Valid and Reliable Measurement of PF* | Method of PF measurement is adequately valid and reliable to limit misclassification bias (e.g., may include  relevant outside sources of information on measurement properties, also characteristics, such as blind measurement and limited reliance on recall). |  |  |  |
| --- | --- | --- | --- | --- |
|  | Continuous variables are reported or appropriate cut-points (i.e., not data-dependent) are used. | If continuous value or 2.0/4.0 mmol/L (defined in the methods section) are used = yes |  |  |
| *Method and Setting of PF Measurement* | The method and setting of measurement of PF is the same for all study participants. |  |  |  |
| *Proportion of data on PF available for*  *analysis* | Adequate proportion of the study sample has complete data for PF variable. | Loss to follow up > 10% = partial, >20% = no |  |  |
| *Method used for missing data* | Appropriate methods of imputation are used for missing 'PF' data. | NOT INCLUDED IN OUR RISK OF BIAS ASSESSMENT |  |  |
| **PF Measurement Summary** | ***PF* is adequately measured in study participants to sufficiently limit potential bias.** |  |  |  |
|  | | | | |
| **4. Outcome**  **Measurement** | **Goal: To judge the risk of bias related to the measurement of outcome (differential measurement of outcome related to the baseline level of PF).** |  |  |  |
| *Definition of the Outcome* | A clear definition of outcome is provided, including duration of follow-up and level and extent of the outcome construct. |  |  |  |
| *Valid and Reliable Measurement of Outcome* | The method of outcome measurement used is adequately valid and reliable to limit misclassification bias (e.g., may include relevant outside sources of information on measurement properties, also characteristics, such as blind measurement and confirmation of outcome with valid and reliable test). |  |  |  |
| *Method and Setting of Outcome*  *Measurement* | The method and setting of outcome measurement is the same for all study participants. |  |  |  |
| **Outcome Measurement Summary** | ***Outcome of interest* is adequately measured in study participants to sufficiently limit potential bias.** |  |  |  |
|  | | | | |
| **5. Study Confounding** | **Goal: To judge the risk of bias due to confounding (i.e. the effect of PF is distorted by**  **another factor that is related to PF and outcome).** |  |  |  |
| *Important Confounders Measured* | All important confounders, including treatments (key variables in conceptual model: LIST), are measured. | Age & sex & comorbidity = yes, Age & sex = partly |  |  |
| *Definition of the confounding factor* | Clear definitions of the important confounders measured are provided (e.g., including dose, level, and duration of exposures). | NOT INCLUDED IN OUR RISK OF BIAS ASSESSMENT |  |  |
| *Valid and Reliable Measurement of Confounders* | Measurement of all important confounders is adequately valid and reliable (e.g., may include relevant outside sources of information on measurement properties, also characteristics, such as blind measurement and  limited reliance on recall). | Unsure, if no confounders are listed |  |  |
| *Method and Setting of Confounding*  *Measurement* | The method and setting of confounding measurement are the same for all study participants. |  |  |  |
| *Method used for missing data* | Appropriate methods are used if imputation is used for missing confounder data. | NOT INCLUDED IN OUR RISK OF BIAS ASSESSMENT |  |  |
| *Appropriate Accounting for Confounding* | Important potential confounders are accounted for in the study design (e.g., matching for key variables, stratification, or initial assembly of comparable groups). | NOT INCLUDED IN OUR RISK OF BIAS ASSESSMENT |  |  |
|  | Important potential confounders are accounted for in the analysis (i.e., appropriate adjustment). | Unsure, if no confounders are listed |  |  |
| **Study Confounding Summary** | **Important potential confounders are appropriately accounted for, limiting potential bias with respect**  **to the relationship between *PF* and *outcome* .** |  |  |  |
|  | | | | |
| **6. Statistical Analysis**  **and Reporting** | **Goal: To judge the risk of bias related to the statistical analysis and presentation of results.** |  |  |  |
| *Presentation of analytical strategy* | There is sufficient presentation of data to assess the adequacy of the analysis. | A table of the outcome data is presented OR an exact result/value are presented in the text => yes |  |  |
| *Model development strategy* | The strategy for model building (i.e., inclusion of variables in the statistical model) is appropriate and is based on a conceptual framework or model. | NOT INCLUDED IN OUR RISK OF BIAS ASSESSMENT |  |  |
|  | The selected statistical model is adequate for the design of the study. | Using a mean value for lactate measurements is wrong, if comparing means is the final analysis presented => no |  |  |
| *Reporting of results* | There is no selective reporting of results. |  |  |  |
| **Statistical Analysis and Presentation**  **Summary** | **The statistical analysis is appropriate for the design of the study, limiting potential for presentation of**  **invalid or spurious results.** |  |  |  |

**Section 2:** Risk of Bias Assessment in Objective 2

In Objective 2, the cohort studies were assessed using the ‘Newcastle-Ottawa scale (NOS) for assessing the quality of nonrandomized studies’ modified to our primary review questions. The NOS has three domains. Our modified tool is shown below. We assigned an overall Risk of Bias (RoB) using the following criteria:

- Low risk: 3 or 4 stars (★) in the selection domain AND 1 or 2 stars in the comparability domain AND 2 or 3 stars in the outcome domain;
- Some concerns: 2 stars in the selection domain AND 1 or 2 stars in the comparability domain AND 2 or 3 stars in the outcome domain;
- High risk: 0 or 1 star in the selection domain OR 0 stars in the comparability domain OR 0 or 1 star in the outcome domain.

Our modified RoB tool:

**NEWCASTLE - OTTAWA QUALITY ASSESSMENT SCALE COHORT STUDIES**

Note: A study can be awarded a maximum of one star for each numbered item within the Selection and Outcome categories. A maximum of two stars can be given for Comparability

# Selection

1. Representativeness of the exposed cohort
   1. truly representative of the average prehospital patient_ (describe) in the community ★
   2. somewhat representative of the average prehospital patient in the community ★
   3. selected group of users eg nurses, volunteers
   4. no description of the derivation of the cohort
2. Selection of the non exposed cohort
   1. drawn from the same community as the exposed cohort ★
   2. drawn from a different source
   3. no description of the derivation of the non exposed cohort
3. Ascertainment of exposure
   1. secure record (eg surgical records) ★
   2. structured interview ★
   3. written self report
   4. no description
4. Demonstration that outcome of interest was not present at start of study
   1. yes ★
   2. no

# Comparability

1. Comparability of cohorts on the basis of the design or analysis
   1. study controls for _illness severity (select the most important factor) ★
   2. study controls for any additional factor ★ (This criteria could be modified to indicate specific control for a second important factor.)

# Outcome

1. Assessment of outcome
   1. independent blind assessment ★
   2. record linkage ★
   3. self report
   4. no description
2. Was follow-up long enough for outcomes to occur
   1. yes (select an adequate follow up period for outcome of interest) ★
   2. no
3. Adequacy of follow up of cohorts
   1. complete follow up - all subjects accounted for ★
   2. subjects lost to follow up unlikely to introduce bias - small number lost - > 90 % (select an adequate %) follow up, or description provided of those lost) ★
   3. follow up rate < _90_% (select an adequate %) and no description of those lost
   4. no statement

**Section 3:** Objective 3

***Objective 3: The clinicians’ opinions on acute prehospital lactate measurement in early decision-making***

**Results:**

We included four studies. Three studies investigated this subject using questionnaires, and one used semi-structured qualitative interviews. One study was conducted in out-of-hours primary care and differed substantially from the others in prehospital emergency services settings. Two studies investigated the opinions on a panel of tests, including a lactate measurement and two studies examined the use of lactate measurements alone. See Table 1 here in Supplementary Files.

Objective 3 posed a high risk of selective reporting of results. One of the studies was only reported as a conference abstract.

**Discussion:**

Three out of four studies investigated clinicians’ opinions on acute prehospital blood lactate measurement as a valuable tool in early decision-making. These three studies were conducted in the prehospital emergency service. One of the studies investigated the clinicians’ perceived benefit of arterial blood gases. The results from this study are not solely based on lactate levels; precautions must be taken when interpreting this study’s findings.

One study investigated a blood test panel in out-of-hours primary care. The investigators discovered several hurdles during the study, and a test was only used in 2% of the possible cases. The lactate level was included in the test panel in 20.4% (11/54) of the cases in this study. Hence, the findings in this study may not reflect the clinicians’ opinions on prehospital lactate measurements alone but likely on the other prehospital blood tests available as well. We cannot compare in-hospital settings, as we did not identify studies investigating the clinicians’ opinions on in-hospital lactate measurement in early decision-making.

**Table 1.** Objective 3. Studies investigating clinicians’ opinions on acute prehospital blood lactate measurement as a valuable tool in early decision-making.

| First author,  Year,  Reference | Study design | Setting | Patients | Participants (N) | Measurement | Lactate measurement standard care | Results |
| --- | --- | --- | --- | --- | --- | --- | --- |
| Alves,*  2020,  [48] | Questionnaire | Prehospital Emergency Service,  France | Sepsis/ septic shock | 60 (60% of invited participants) | Lactate level | Partly (25%) | 57/60 participants (95%) responded that lactate measurement helps assess illness severity in sepsis/septic shock. |
| Hayward & Dixon,  2019 & 2021  [49] & [50] | Semi-structured qualitative interviews | Out-of-hours Primary care,  England | Mixed | 16  (19 inter-views) | A panel of blood tests. 20.4% of tests included a lactate level | No | Reasons for low uptake of testing (2% of potential cases) were: Time pressure, practical challenges, the uncertain clinical value of testing, and potential medicolegal risks.  Pro: Clinical confidence. Support communication with patients and professionals. |
| Younger,  2014,  [46] | Questionnaire | Prehospital Emergency Service,  England | Sepsis | 7 | Lactate level | No | 6/7 participants (86%) responded that lactate measurement increased their confidence in sepsis diagnosis.  6/7 participants (86%) responded that they felt patients would benefit from lactate measurement. |
| Zwisler,  2019,  [47] | Questionnaire (included in a Randomized controlled trial) | Prehospital Emergency Service, Denmark | Critically ill (Glasgow Coma Score < 13) | 13 | Arterial blood gas, including a lactate level | Yes | The clinicians answered a questionnaire after treating each patient (220 cases). The perceived benefit of arterial blood gas analysis was crucial in 20 cases (9.0%), of major importance in 68 cases (30.6%), of moderate importance in 61 cases (27.5%), minor importance in 42 cases (18.9%), and not important in 28 cases (12.6%). |

Studies are listed alphabetically by author name. Unless otherwise stated, all data was extracted from the primary study report of each study. *The study was reported as a conference abstract, and we could not retrieve additional information.
